# Cytochrome P450-derived metabolites of docosahexaenoic acid or arachidonic acid enhance contractility of cultured rat cardiomyocytes: a pilot study

**DOI:** 10.1007/s10047-026-01552-z

**Published:** 2026-04-03

**Authors:** Hikari Kawakami, Daisuke Sato, Masaki Sazuka, Saki Tsuchida, Atsuyoshi Nishina, Takao Nakamura, Masataka Kusunoki, Kazuhiko Tsutsumi, Zhonggang Feng

**Affiliations:** 1 Department of Biochemical Engineering, Graduate School of Science and Engineering, Yamagata University, 4-3-16 Jonan, Yonezawa, 992-8510 Japan; 2 Faculty of Health and Nutrition, Yamagata Prefectural Yonezawa University of Nutrition Sciences, Yonezawa, Japan; 3 Department of Health and Nutrition, Tokai Gakuen University, Nagoya, Japan; 4 Yamagata University, Yamagata, Japan; 5 Apollon Nagoya Exercise Therapy Center for Diabetes, Akishima Clinic, Nagoya, Japan; 6 Department of Mechanical Engineering, Graduate School of Science and Engineering, Yamagata University, Yonezawa, Japan

**Keywords:** Cardiomyocyte, Contractility, Cytochrome P450-derived metabolite, Docosahexaenoic acid, Arachidonic acid

## Abstract

Regenerative medicine is perceived as a new treatment for severe heart disease. However, cell-based regenerative therapy for cardiac tissue primarily contributes through its paracrine effect, with limited direct impact on contractile force. We have previously found that docosahexaenoic (DHA) and arachidonic (AA) acid could improve the contractility of cultured cardiomyocytes. On the other hand, the beneficial effect of their metabolites produced by cytochrome P450 (CYP) has not been clarified yet because CYPs are abundantly expressed in the liver. In the present study, we evaluated the effects of CYP-derived metabolite of DHA or AA on contractility of cultured rat cardiomyocytes. The cardiomyocytes were cultured in the medium which were supplemented each metabolite for 4 days, and then we evaluated contractile fraction, beat rate, and contraction velocity under a microscope. In addition, we fabricated collagen gel-based myocardial tissue, cultured them for 24 h in the metabolites-containing medium, and measured the contractile force generated by the tissue. 19,20-epoxydocosapentaenoic acid (EpDPE) enhanced both contractile fraction and contraction velocity of cardiomyocytes, whereas 22-hydroxydocosahexaenoic and 8,9-eicosatrienoic acids increased contractile fraction only, and 18- and 20-hydroxyeicosatetraenoic acids increased contraction velocity. Furthermore, 19,20-EpDPE tended to increase the contractile force of myocardial tissue, whereas none of the other metabolites produced a comparable effect. These results indicate that some DHA and AA metabolites can improve contractile fraction as well as contraction velocity of cardiomyocytes in culture; however, an appreciable contribution to contractile force within 24 h is not anticipated.

**Supplementary Information:**

The online version contains supplementary material available at 10.1007/s10047-026-01552-z.

## Introduction

Reconstructed myocardial tissue in vitro is inferior to its counterpart in vivo in mechanical properties, even though regenerative modality in cardiological medicine is attracting intense attention as a new treatment for serious heart diseases [1, 2]. Recently, experimental treatments involving the transplantation of stem cell-derived cardiomyocytes into animal and human hearts have shown promising results, reporting the potential for functional recovery in hearts compromised by conditions such as heart failure. A significant portion of this functional regeneration is attributed to the paracrine effects, such as angiogenesis, induced by the transplanted cells, suggesting that the direct contribution of the transplanted cells themselves to contractile force is relatively small [2, 3]. For instance, it is reported that the twitch stress generated in three-dimensional myocardial tissue engineered from fetal rat cardiomyocytes by using collagen scaffold is only 2 kPa, while the stress in native myocardium is > 20 kPa [4].

Fatty acids are classified into saturated fatty acids (SFAs), monounsaturated fatty acids (MUFAs), and polyunsaturated fatty acids (PUFAs), and these are known to be major energy sources in the myocardium. However, excessive intake of SFAs can increase the risk of heart disease [5], and although there is no consensus on MUFAs, there are several reports that they are associated with cardiovascular disease [6, 7]. PUFAs, also known as essential fatty acids, are not only used as an energy source but also play a role in the regulation of cell functions via modulation of gene transcription [8–10]. Notably, fatty acids including PUFAs as supplemented to culture media were able to improve cell maturity of cultured cardiomyocytes [11–13]. Mammals can biosynthesize only SFA and MUFA because they do not have desaturation enzyme necessary for making a double bond at n-3 or n-6 binding site. In spite of the facts, the conventional medium used in cell culture contains very little lipids. Therefore, the cultured cardiomyocyte might be under malfunction to generate physiological properties because of insufficiency of PUFAs.

We previously compared the fatty acid composition in cultured rat fetal cardiomyocytes with that in neonatal myocardium and found considerable shortage of n-3 and n-6 PUFAs in cultured cells [14]. In particular, the contents of docosahexaenoic acid (DHA), and arachidonic acid (AA) were significantly lower than those in the neonatal tissue. Therefore, we added DHA or AA alone in the medium to clarify the effects on cultured cardiomyocytes, and demonstrated that DHA or AA elevated the contractile fraction (CF) which calculated from the area change caused by beating and the mRNA expressions of *N-cadherin* and *Connexin 43*, which are involved in cell adhesion [15].

Dietary DHA and AA are metabolized mainly in the liver by enzymes such as cyclooxygenase, lipoxygenase, and cytochrome P450 (CYP) in vivo [16]. In particular, CYPs are present in various organs and produce a wide variety of metabolites [17, 18]. PUFAs and their metabolites have an aspect as lipid mediators, and the metabolites have beneficial effects on cardiomyocyte function, such as protecting cells from Ca^2+^ overload that causes cell impairment and hypertrophy through cAMP-dependent signaling [19]. However, CYPs are abundantly expressed in the liver, the metabolic capacity of CYPs in myocardium is known to be relatively lower [20]. This suggests that the insufficient supply of CYP-derived PUFA metabolites in cardiomyocyte culture could be one of the contributing factors to the malfunction on contractility. However, there have been few reports on the effects of supplementation of PUFA metabolites on contractile ability in cultured cardiomyocytes.

In the present study, we cultured cardiomyocytes under supplementation of major CYP-derived metabolites of DHA or AA to investigate the effects of the metabolites on contractile ability of cultured cardiomyocytes. In addition, we also conducted a feasibility study to determine whether these metabolites enhance contractile force generated by myocardial tissue.

## Materials and methods

### Chemicals

All the metabolites derived from DHA and AA were purchased from Cayman Chemical (Ann Arbor, MI). The code numbers for the respective compounds are as follows: (±)7,8-epoxydocosapentaenoic acid (EpDPE): 10465, (±)10,11-EpDPE: 10471, (±)19,20-EpDPE: 10175, or 22-hydroxydocosahexaenoic acid (HDoHE): 19321, (±)8,9-epoxyeicosatrienoic acid (EET): 50351, (±)11,12-EET: 50511, (±)14,15-EET: 50651, (±)18-hydroxyeicosatetraenoic acid (HETE): 10010638, 19(S)-HETE: 10007766, or 20-HETE: 90030. The purity of each metabolite was guaranteed to be ≥ 90%.

## Cardiomyocyte primary culture

The research was conducted in accordance with the Guide for the Care and Use of Laboratory Animals as adopted and promulgated by the United States National Institutes of Health, and the animal handling and experimental methods used in this study were approved in advance by the Yamagata University Animal Experiment Committee (No. R6050).

Female Wistar rats, obtained from an in-house breeding colony, were housed in a room maintained at 21 ± 1 °C and 12-h light/dark cycle, and allowed free access to chow and tap water. The details of the harvest and primary culture of rat embryonic cardiomyocytes are described elsewhere [14]. In brief, 18 days after impregnation, the rats were anesthetized with 4% isoflurane, and the abdominal artery were cut at laparotomy. Fetal rats were taken out immediately and their ventricles were harvested by thoracotomy after lumber fracture. The ventricles were digested to isolate cardiomyocytes with 0.1% type I collagenase and 0.1% D-glucose in phosphate buffered saline (Sigma-Aldrich, St. Louis, MO) over five cycles, each lasted for 8 min.

The cells were cultured at the seeding density of 1 million per 60 mm plastic dish for 7 days with DMEM/F12-Ham (Sigma-Aldrich) containing 10% FBS (Nichirei Biosciences, Tokyo, Japan), 1% penicillin-streptomycin (Sigma-Aldrich), and 260 mU/mL insulin (Humulin R; Eli Lilly Japan, Tokyo, Japan) in a 5%-CO_2_ incubator at 37 °C (day 0). The culture medium was changed firstly 24 h after the cell seeding (day 1) and then every other day.

## Supplementation of DHA- or AA-derived metabolites

Cardiomyocytes isolated from a single litter were assigned to either control or various metabolite concentration experimental groups. Due to the inherent variability in the quantity of obtainable cardiomyocytes, if a deficiency in the number of experiments (n) occurred for specific concentration settings, the n-value is presented as a range.

Three days after the start of cell culture, cardiomyocytes were cultured with supplementation of the following DHA or AA-derived epoxide or hydroxide. We supplemented individually one of four metabolites of DHA: 7,8-EpDPE (*n* = 5), 10,11-EpDPE (*n* = 5), 19,20-EpDPE (*n* = 6), or 22-HDoHE (*n* = 4–9), or one of six metabolites of AA: 8,9-EET (*n* = 5), 11,12-EET (*n* = 5), 14,15-EET (*n* = 5), 18-HETE (*n* = 7), 19-HETE (*n* = 6), or 20-HETE (*n* = 6). Each metabolite was dissolved in dimethyl sulfoxide (DMSO) and supplemented to the medium at the same time as the medium change. Since Naoe et al. reported the concentration of oxylipins in rodent plasma are less than approximately 50 nM [21], the concentration of 22-HDoHE was set to 0.001, 0.01, 0.1, 1, 10, and 100 nM, and the other concentrations were set to 0.01, 0.1, 1, 10, and 100 nM. Control group was the cells cultured with the only addition of DMSO.

## Assessment of the CF and contraction velocity

Four days after the onset of supplementation of DHA or AA-derived metabolites (on day 7), we arbitrarily selected several colonies of beating cardiomyocytes under a microscope and recorded their video image on a personal computer. We calculated CF as shown elsewhere [15, 22] with minor modification. In brief, we arbitrarily selected each beating colony on the video image, and the beating area was approximated by a polygon using ImageJ ver. 1.54d (https://imagej.net/ij/). We calculated each area during a beating cycle, and defined CF as the formula shown below:

$$CF\left( \% \right) = \left( {1 - \frac{{{\mathrm{Minimun~area}}}}{{{\mathrm{Maximum~area}}}}} \right) \times 100$$

The contraction velocity was determined by dividing the moving distance of any one point forming the polygon by the time required for contraction. Beat rate was manually counted for 10 s on the video image, and then the quantity was sextuplicated to obtain beat rate per minute.

### Measurement of mRNA expression

When we found the metabolites elevate the CF, we measured mRNA expressions involved in differentiation and maturation (*Mlc2v*, *Mlc2a*, *Srf*, *Nkx2.5*, and *cTnT*), cell adhesion (*Cdh2* and *Cx43*), heart contraction (*Ryr2*, *Serca2a*, and *Hcn4*), and lipid metabolism (*Cd36*, *Pparα*, *Pparδ*, and *Cox IV*) by real-time PCR according to the instructions provided by the reagent manufacturer. β-actin was used as an internal control. Target genes and their sequences of primers used in the present study are shown in Table S1.

At the day 7, cultured cells were collected, and homogenized in TRIzol^®^ reagent (Thermo Fisher Scientific, Waltham, MA) to extract total RNA. The first strand cDNA was synthesized from 500 ng of total RNA using PrimeScript^®^ RT reagent Kit (Takara Bio, Kusatsu, Japan). The amplification and detection of mRNA was performed with TB Green^®^ Premix Ex Taq™ II (Takara Bio). The thermal cycling program was as follows: 30 s denaturation step at 95 °C, followed by 40 cycles of 5 s denaturation at 95 °C and 30 s annealing at 60 °C. Melting curve analysis was conducted to confirm the amplification products. Gene expression in individual samples was determined using ΔΔCt method. Mean levels in the control group were arbitrarily assigned a value of 1.

## Measurement of the contractile force of cultured cardiomyocytes

**Fig. 1:**
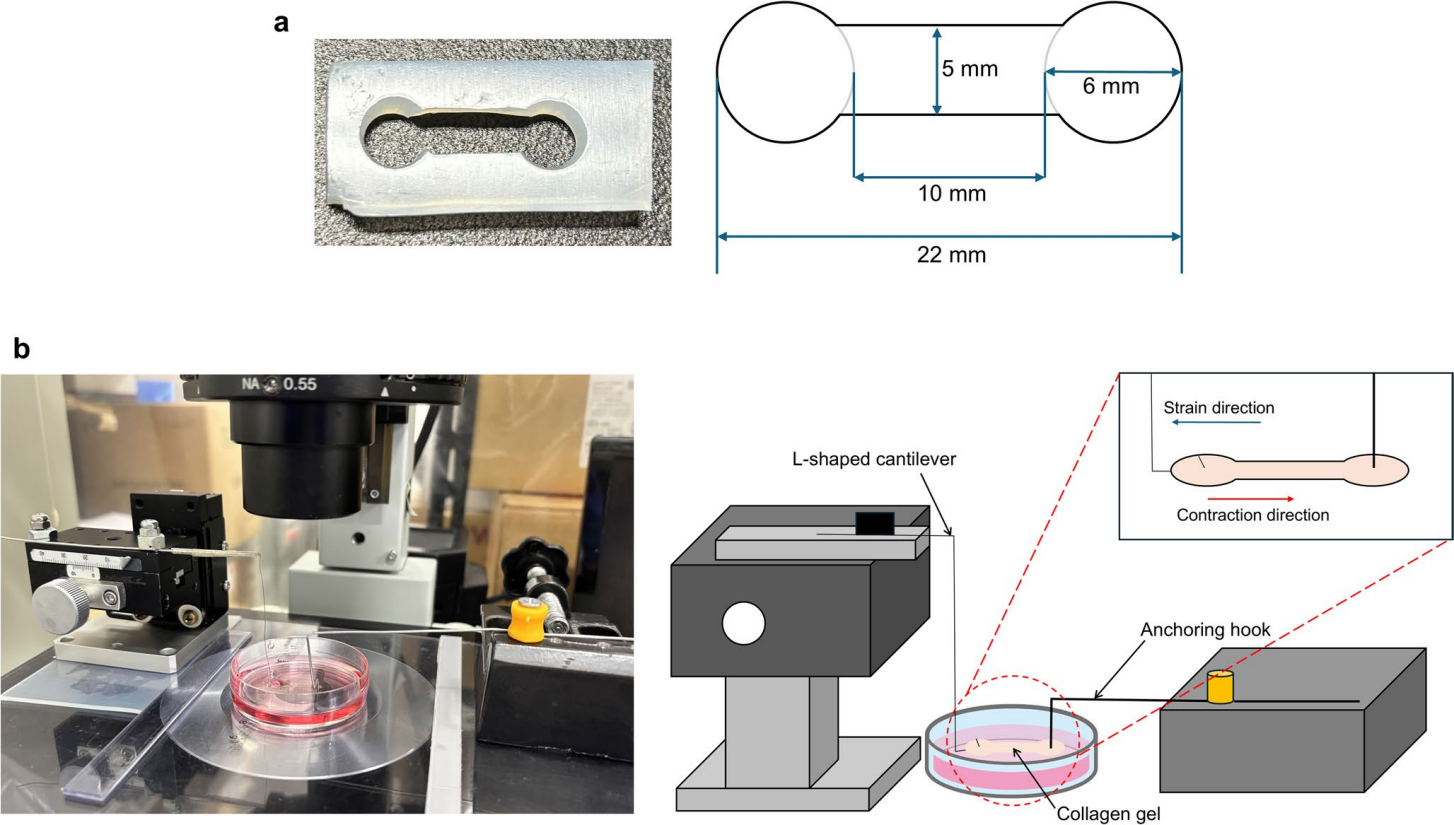
Shape of the silicone rubber mold for collagen gel fabrication (**a**) and overview of measurement device (**b**)

To measure contractile force of cardiomyocyte, we used lab-made vision-based measurement system which is adapted from an earlier study [15]. In brief, we fabricated a dumbbell-shaped collagen gel (10 mm in length, 5 mm in width) with 6 mm-diameter circular grips at each end with casting collagen solution into silicone rubber molds (Fig. 1a). The collagen solution was formed by mixing type I collagen (Corning) with DMEM/F12-Ham and 1 N NaOH to obtain a neutralized solution at 1.5 mg/mL collagen concentration. Each collagen gel had an initial volume of 0.4 mL so that the initial thickness of the gel was 1.0 mm. Gelation was allowed in a 5%-CO_2_, 37 °C incubator for 1 h. After the gelation, 1 mL crosslinker solution, 1-ethyl-3-(3-dimethylaminopropyl) carbodiimide in distilled water (DW) at 50 mM concentration, was added into the mold to modulate the mechanical properties of the gel. The crosslinking was conducted under room temperature for 48 h. After crosslinking, gels were washed with DW and PBS supplemented with 1% penicillin. After washing, the gels were coated with 1 mL solution of Matrigel (Corning) in DMEM/F12-Ham at 10.9 µL/mL volume concentration in an incubator for 1 h to enhance cellular adhesion to the gel. The cardiomyocytes obtained with the aforementioned method were seeded on the collagen gel at a density of 125 × 10^4^ cells/gel, and were cultured with DMEM/F12-Ham containing 10% FBS. Four hours later, we added metabolites which tended to elevate the CF or contraction velocity into the medium.

Twenty-four hours after cell seeding, the tissue was cultured in the medium containing metabolites which tended to elevate the CF or contraction velocity for 24 h (control: *n* = 8; 1 nM 19,20-EpDPE: *n* = 8; 1 nM 20-HDoHE: *n* = 8; 0.1 nM 8,9-EET: *n* = 5; 10 nM 18-HETE: *n* = 6; 0.1 nM 20-HETE: *n* = 6). Twenty-four hours after the culture, spontaneously beating circular cardiomyocyte-collagen gel was installed in the measuring device (Fig. 1b); one end of the gel was anchored with a single stainless-steel hook (1 mm wire diameter), and the opposite end was attached to an L-shaped silver wire cantilever (0.2 mm diameter). The sample was immersed into the culture medium. The displacement of the L-shaped cantilever caused by beating was measured under a microscope at different stretch strains of the cardiomyocyte-collagen gel. The stretch strain was achieved by pulling the silver cantilever at 5, 10, and 15% stretch ratio to the initial side length, respectively. The displacement was calibrated with known weights of 3–8 mg, and the relationship between the weight and displacement was linear (R^2^ > 0.99). Thus, we calculated the contractile force of the cardiomyocyte-collagen gel from the displacement and slope.

### Statistical analysis

Data are expressed as mean ± SE. To obtain dose-response information, multiple comparison tests were performed using one-way analysis of variance (ANOVA) and Williams post hoc test to compare the data in the groups under supplementation of DHA- or AA-derived metabolite with the data in the cells cultured without metabolites (control). The effects of individual metabolites on contractile force were compared with the control group by one-way ANOVA followed by Dunnett’s post hoc test. To detect significant difference in the mRNA expression between groups, we used unpaired t-test. *P* < 0.05 was regarded as significance.

## Results

### Supplementation of DHA-derived metabolites

On day 7 of culture, there was no significant difference in the CF compared to the control group at each concentration under 0.01–100 nM 7,8-EpDPE or 10,11-EpDPE supplementation while the CF tended to be dose-dependently higher and became significant (*P* < 0.05) under 1 nM 19,20-EpDPE supplementation. In the cells under 22-HDoHE supplementation, the CF was significantly higher than that in the control group at all examined concentration (Table 1).

**Table 1:** Parameters of contractile function of cardiomyocytes at the end of culture period (on day 7) under supplementation of docosahexaenoic acid-derived metabolites

	*n*			Concentration (nM)					
			Control (0 nM)	0.001 nM	0.01 nM	0.1 nM	1 nM	10 nM	100 nM
	Contractile fraction (%)								
7,8-EpDPE	5		8.28 ± 0.64		7.66 ± 0.32	8.66 ± 0.25	7.66 ± 1.52	7.84 ± 0.65	8.76 ± 0.50
10,11-EpDPE	5		6.92 ± 0.92		5.66 ± 0.59	5.84 ± 0.52	6.50 ± 0.63	7.40 ± 1.12	6.62 ± 1.15
19,20-EpDPE	6		6.47 ± 0.78		7.65 ± 0.61	7.48 ± 0.61	8.70 ± 0.53*	7.70 ± 1.52	7.32 ± 1.37
22-HDoHE	4–9		6.93 ± 0.41	8.70 ± 0.73*	9.21 ± 0.61**	8.42 ± 0.42**	9.28 ± 0.51**	9.03 ± 0.50**	8.42 ± 0.63**
	Beat rate (bpm)								
7,8-EpDPE	5		65 ± 9		84 ± 28	96 ± 27	89 ± 24	71 ± 13	64 ± 10
10,11-EpDPE	5		93 ± 20		84 ± 8	104 ± 20	106 ± 21	89 ± 13	98 ± 12
19,20-EpDPE	6		149 ± 34		127 ± 12	132 ± 16	103 ± 14	133 ± 29	139 ± 29
22-HDoHE	4–9		115 ± 15	78 ± 18	88 ± 14	124 ± 25	97 ± 14	112 ± 21	109 ± 22
	Contraction velocity (ratio to the control group)								
7,8-EpDPE	5				0.98 ± 0.07	1.19 ± 0.15	1.22 ± 0.11	0.97 ± 0.10	0.93 ± 0.14
10,11-EpDPE	5				0.70 ± 0.18	0.83 ± 0.19	0.87 ± 0.16	0.86 ± 0.14	0.86 ± 0.23
19,20-EpDPE	6				1.56 ± 0.28*	1.26 ± 0.20	1.61 ± 0.17**	1.38 ± 0.22	1.40 ± 0.22
22-HDoHE	4–9			1.04 ± 0.04	1.30 ± 0.22	1.20 ± 0.17	1.17 ± 0.15	1.16 ± 0.18	1.09 ± 0.22

Mean ± SE. **P < 0.01, *P < 0.05 versus control group

We detected no significant difference between the control and metabolite-supplemented groups in the beat rate.

The contraction velocity tended to be higher than those in the control group only under 19,20-EpDPE supplementation, and the differences were significant under 0.01 nM (*P* < 0.05) or 1 nM (*P* < 0.01) supprementation (Table 1).

Regarding mRNA expression, only *Mlc2v* and *Hcn4* were significantly lower (*Mlc2v*: *P* < 0.01 and *Hcn4*: *P* < 0.05) in 1 nM 19,20-EpDPE-supplemented cells than in the control group (Figs. S1 and S2) whereas there was no significant difference in 22-HDoHE-supplemented groups in all examined genes (Figs. S1, S2, S3).

### Supplementation of AA-derived metabolites

On day 7 of culture under EET supplementation, the CF was significantly higher (*P* < 0.05) in the 8,9-EET-treated cells than in control group under 0.1–10 nM supplementation. In contrast, the fraction in the 100 nM 14,15-EET-treated cells was significantly lower (*P* < 0.05) (Table 2). No significant difference in the CF was detected under 11,12-EET, 18-HETE, 19-HETE, and 20-HETE supplementation.

**Table 2:** Parameters of contractile function of cardiomyocytes at the end of culture period (on day 7) under supplementation of arachidonic acid-derived metabolites

	*n*		Concentration (nM)				
		Control (0 nM)	0.01 nM	0.1 nM	1 nM	10 nM	100 nM
		Contractile fraction (%)					
8,9-EET	5	6.98 ± 0.76	8.20 ± 0.85	9.52 ± 0.57*	9.32 ± 0.90*	9.48 ± 1.40*	8.80 ± 1.20
11,12-EET	5	8.96 ± 0.44	8.38 ± 0.49	9.52 ± 0.65	9.06 ± 1.18	9.62 ± 1.00	9.85 ± 1.09
14,15-EET	5	6.80 ± 0.31	6.06 ± 0.40	7.60 ± 1.04	5.78 ± 0.85	6.38 ± 0.60	4.92 ± 0.58*
18-HETE	7	6.56 ± 0.82	6.40 ± 0.90	7.87 ± 0.92	7.40 ± 1.13	7.66 ± 0.97	6.91 ± 1.31
19-HETE	6	7.60 ± 0.83	8.57 ± 0.78	7.65 ± 0.58	9.20 ± 0.64	8.68 ± 0.82	7.92 ± 0.54
20-HETE	6	7.98 ± 0.47	8.38 ± 0.64	8.98 ± 1.11	8.67 ± 0.60	9.38 ± 1.38	8.43 ± 0.39
		Beat rate (bpm)					
8,9-EET	5	117 ± 27	87 ± 18	89 ± 13	80 ± 12	80 ± 11	103 ± 21
11,12-EET	5	109 ± 19	121 ± 37	107 ± 21	107 ± 20	83 ± 10	109 ± 22
14,15-EET	5	126 ± 16	102 ± 10	105 ± 9	128 ± 13	149 ± 16	162 ± 21
18-HETE	7	101 ± 23	123 ± 29	100 ± 25	103 ± 18	115 ± 21	116 ± 31
19-HETE	6	98 ± 15	77 ± 8	108 ± 15	99 ± 21	92 ± 13	118 ± 28
20-HETE	6	101 ± 18	83 ± 10	85 ± 8	75 ± 9	98 ± 16	87 ± 13
		Contraction velocity (ratio to the control group)					
8,9-EET	5		1.24 ± 0.26	1.09 ± 0.14	1.18 ± 0.20	1.11 ± 0.08	0.94 ± 0.11
11,12-EET	5		0.95 ± 0.08	0.99 ± 0.13	0.79 ± 0.06	0.83 ± 0.10	0.98 ± 0.24
14,15-EET	5		0.90 ± 0.15	0.94 ± 0.10	0.83 ± 0.08	0.83 ± 0.10	0.82 ± 0.10
18-HETE	7		1.07 ± 0.12	1.19 ± 0.11	1.34 ± 0.14*	1.40 ± 0.17**	1.25 ± 0.17
19-HETE	6		0.93 ± 0.15	0.85 ± 0.09	1.13 ± 0.18	1.03 ± 0.24	1.04 ± 0.18
20-HETE	6		1.11 ± 0.07	1.63 ± 0.29**	1.31 ± 0.19*	1.15 ± 0.12	1.13 ± 0.10

Mean ± SE. **P < 0.01, *P < 0.05 versus control group

We found no significant difference in the beat rate between the metabolite-supplemented cells and control group as seen in the DHA-derived metabolites.

The contraction velocity was significantly higher in 1–10 nM 18-HETE- or 0.1-1 nM 20-HETE-supplemented cells than in their control (Table 2). However, the other four metabolites did not affect the velocity.

AA-derived metabolites did not affect the expression of any genes except for PPARs (Figs. S1, S2, S3). *Pparδ* expression was significantly lower (*P* < 0.05) in the 0.1 nM 8,9-EET-supplemented cells than in control cells. On the other hand, significantly higher expression of *Pparα* was observed only under 10 nM 18-HETE supplementation (*P* < 0.05) (Fig. S3).

### Contractile force

**Fig. 2:**
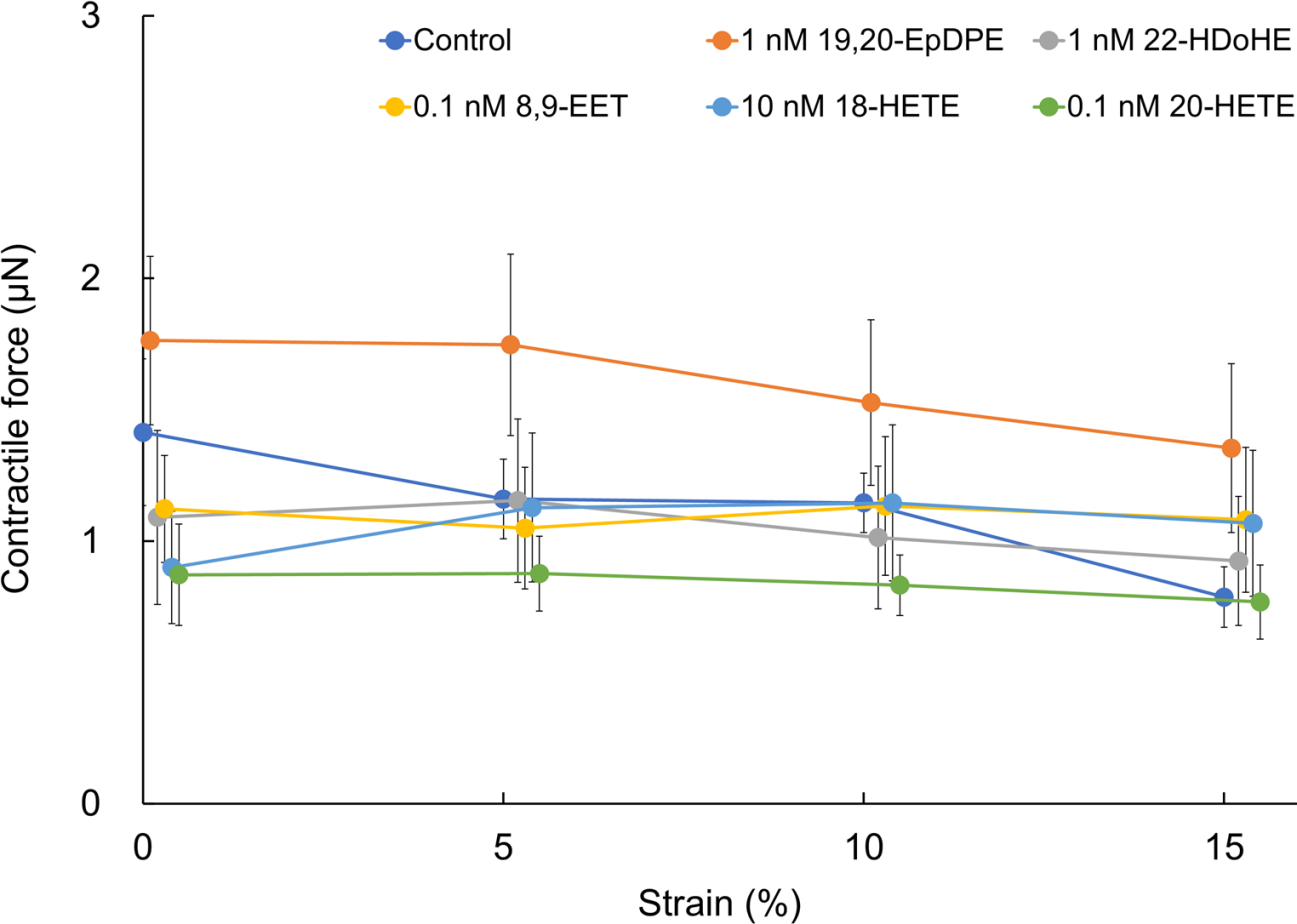
Contractile force generated by embryonic rat cardiomyocytes in cardiomyocyte-collagen gel (control: *n* = 8; 1 nM 19, 20-EpDPE: *n* = 8; 1 nM 20-HDoHE: *n* = 8; 0.1 nM 8, 9-EET: *n* = 5; 10 nM 18-HETE: *n* = 6; 0.1 nM 20-HETE: *n* = 6). Data are expressed as mean ± SE

Twenty-four hours after the supplementation of 1 nM 19,20-EpDPE, the contractile force of collagen gel-based myocardial tissue tended to be higher than the control group at all stretch ratio (not significant). On the other hand, we did not observe significant differences in the contractile force at all stretch ratio under 1 nM 22-HDoHE, 0.1 nM 8,9-EET, 10 nM 18-HETE, or 0.1 nM 20-HETE supplementation (Fig. 2). To illustrate the data trends, *p*-values for all comparisons are provided in Table S2.

## Discussion

As a result of supplementation of 7,8-EpDPE or 10,11-EpDPE, there was no significant difference in the CF, beat rate, and contraction velocity compared to those in the control group. These results suggest that these EpDPEs might not significantly affect the contractility of cultured cardiomyocytes. While 19,20-EpDPE also had no effect on the beat rate and contraction velocity, the CF elevated under 1 nM supplementation.

Supplementation of 22-HDoHE elevated the CF without changing in beat rate and contraction velocity at all examined doses. Several studies suggest the beneficial effects of EpDPEs on cardiovascular function [23] whereas the effects of EpDPEs and HDoHEs in cardiomyocytes have not been documented well. To the best of our knowledge, this is first report regarding the effects of 19,20-EpDPE and 22-HDoHE on contractility of in vitro cardiomyocytes. However, these treatments showed no statistically significant effect on mRNA expression involved in differentiation, maturation, Ca^2+^ transport, and metabolism. Hence, the mechanism underlying the improvement of contractile performance was not clarified from the evaluation of mRNA expression.

While 19,20-EpDPE tended to elevate the contractile force, 22-HDoHE did not affect this function. Not only CF but also contraction velocity may be crucial for improving the contractile force of myocardial tissue. In the present study, cells were exposed to metabolites for 24 h. Extending the exposure time further might lead to a more pronounced effect of these metabolites on contractile force.

In the case of supplementation of EET, 0.1–10 nM 8,9-EET elevated the CF, suggesting that 8,9-EET can improve contractility of cultured cardiomyocytes. By contrast, 100 nM 14,15-EET resulted in suppression of the CF, implying that large amount of this metabolite may inhibit contractile function of in vitro cardiomyocytes.

Under supplementation of HETE, no significant effect was observed on the CF and beat rate. Regarding the contraction velocity, 1–10 nM 18-HETE and 0.1-1 nM 20-HETE enhanced the velocity whereas 19-HETE had no significant effect. Besides, *Pparα* expression was elevated with 10 nM 18-HETE. Recently, it has been reported that PPARα agonist fenofibrate promotes maturation of human pluripotent stem cell-derived cardiomyocytes [24]. In addition, several oxylipins derived from linoleic acid, which is one of n-6 PUFAs, can play as agonists of PPARα and γ [25, 26]. Although the detailed mechanism was not clear in the present study, 18-HETE, which is also derived from n-6 PUFA, may contribute to the cell maturation via direct or indirect pathway. Since 20-HETE stimulates L-type Ca^2+^ channel [27], the enhancement of contraction velocity may be attributed to Ca^2+^ signaling. Because the expression levels of *Ryr2* and *Serca2a* in the present study cannot be consistent with this mechanism, protein expression analysis and calcium imaging will be necessary in the future.

Regarding contractile force, AA-derived metabolites did not contribute to the improvement of this parameter. It may be that either CF or contraction velocity alone does not affect the improvement of contractile force of myocardial tissue. Based on the results of this study, supplementation with DHA- and AA-derived fatty acid metabolites may have only minimal effects on contractile force, at least over a 24-h period. In the present study, including the results of DHA-derived metabolites, we did not observe the biphasic feature of the cardiomyocyte contractile force with the passive stretch strain. This feature is regarded as the fundamental at the cellular level for the cardiac Frank-Starling law. The loss of the biphasic feature in contractile force may implicate the deviation of myofilament structure in the cardiomyocytes under the culture conditions from in vivo structure. The myocardial tissue was constructed under free culture conditions without adjusting the orientation of the cardiomyocytes, which may have influenced the results.

In conclusion, 19,20-EpDPE, 22-HDoHE, and 8,9-EET improved contractility, while 19,20-EpDPE, 18-HETE, and 20-HETE contributed to elevation in the contraction velocity. However, the mechanisms of these improvements cannot be explained by the marker gene measurements. Additionally, the previous study resulted in no improvement of the contractile force of collagen gel-based myocardial tissue while 19,20-EpDPE tended to increase the force.

One limitation of the present study is that we compared only the effects of different PUFA metabolites. To accurately quantify their effects on the beating function of cardiomyocytes, positive controls such as DHA or AA are necessary. In addition, the present study also served as a feasibility study to investigate whether PUFA metabolites can enhance the contractile force of myocardial tissue; therefore, the exposure durations to PUFA metabolites used here and the resulting contractile force data should be regarded as preliminary. Regarding mRNA expression, because the primary objective of this study was to identify metabolites and their concentrations that maximize contractile performance, we examined the effects of metabolites on expression of functional marker genes only at selected metabolite concentrations. To optimize dosing, it will be necessary to examine the dose-response relationship between metabolite concentrations added to the medium and expression of functional markers. It will also be necessary to optimize the duration of metabolite treatment, which can affect marker gene expression.

Since a variety of PUFA metabolites are produced simultaneously in vivo, it is possible that their interactions regulate the contractility. Considering that mature cardiomyocytes utilize fatty acid as an energy source, supplementation of not only metabolites but also fatty acids including SFA and MUFA in the medium may further integrally lead to improvement of the contractility. In fact, Yoshida et al. reported that these fatty acids could improve the contractile force of human induced pluripotent stem cell-derived cardiac tissue [28].

We recently reported that PUFAs can elevate contractility of myocardial tissue derived from human pluripotent stem cells [22]. Thus, in further study, it may be important to evaluate the effect of the metabolites on the contractile force of human cardiomyocytes. Moreover, to fully elucidate the cellular responses, detailed morphological and histological evaluations are indispensable.

## Supplementary Information

Below is the link to the electronic supplementary material.

Supplementary Material 1

## Data Availability

Data will be made available on request.
