# Supplementary material for: Cytochrome P450-derived metabolites of docosahexaenoic acid or arachidonic acid enhance contractility of cultured rat cardiomyocytes: a pilot study
Source: J Artif Organs. 2026 Apr 3;29(2):25. doi: 10.1007/s10047-026-01552-z (PMC13048951; doi:10.1007/s10047-026-01552-z)
Supplement: Supplementary file 1 — Supplementary Material 1 [file 10047_2026_1552_MOESM1_ESM.docx]

**Table S1**: Sequences of primers used for real-time PCR

| Gene | Primer sequence (5′–3′) | Product length (bp) | RefSeq ID |
| --- | --- | --- | --- |
| *Mlc2v* | Forward: aaagaggctccaggtccaaat  Reverse: cctctctgcttgcgtggtta | 177 | XM 039089640 |
| *Mlc2a* | Forward: gctgcattgaccagaacaga  Reverse: cgtgaggaagacggtgaagt | 281 | BC161995 |
| *Srf* | Forward: gggcatttgggtggcttt  Reverse: tcactcgccctggctctatc | 196 | NM 001109302 |
| *Nkx2.5* | Forward: accctcgggcggataagaa  Reverse: gacaggtaccgctgttgcttga | 178 | NM 053651 |
| *cTnT* | Forward: agaggactccaaacccaagc  Reverse: attgcgaatacgctgctgtt | 250 | NM 012676 |
| *Cx43* | Forward: agcaagctagcgagcaaaac  Reverse: gagttcatgtccagcagcaa | 152 | X06656 |
| *Cdh2* | Forward: ctgccatgaccttctacgga  Reverse: gggtctgtcaggatggcaaa | 163 | NM 031333 |
| *Cd36* | Forward: atgagactgggaccatcggc  Reverse: caacaaacatcactactccaacacc | 123 | NM 031561 |
| *Pparα* | Forward: ccatacaggagagcagggatt  Reverse: ccaccatttcagtagcagga | 146 | NM 013196 |
| *Pparδ* | Forward: gcagcctcaacatggagtg  Reverse: gttgcggttcttcttctgga | 167 | NM 013141 |
| *Cox IV* | Forward: gcggaatgttggctaccagggc  Reverse: atgtgcccgaaggcacaccg | 78 | BC084719 |
| *Ryr2* | Forward: taacctaccaggccgtggat  Reverse: gctgcgatctggataagttcaa | 114 | XM 063276763 |
| *Serca2a* | Forward: ctggccgacgacaacttctc  Reverse: tgaggtagcggatgaactgctt | 111 | AF043106 |
| *Hcn4* | Forward: ctccaggagcctgcagttctc  Reverse: gcaagaagtgctccaggagtc | 228 | NM 021658 |
| *β-actin* | Forward: acggtcaggtcatcactatcg  Reverse: ggcatagaggtctttacggatg | 155 | NM 031144 |

**Table S2** P-values from multiple comparisons for contractile force data

| Metabolite | n | Strain (%) | | | |
| --- | --- | --- | --- | --- | --- |
|  |  | 0 | 5 | 10 | 15 |
| 19,20-EpDPE | 8 | 0.830 | 0.353 | 0.675 | 0.274 |
| 22-HDoHE | 8 | 0.867 | 1.000 | 0.994 | 0.962 |
| 8,9-EET | 5 | 0.943 | 0.999 | 1.000 | 0.886 |
| 18-HETE | 6 | 0.602 | 1.000 | 1.000 | 0.883 |
| 20-HETE | 6 | 0.556 | 0.921 | 0.854 | 1.000 |

P-values shown are from comparisons versus the Control group (n=8).


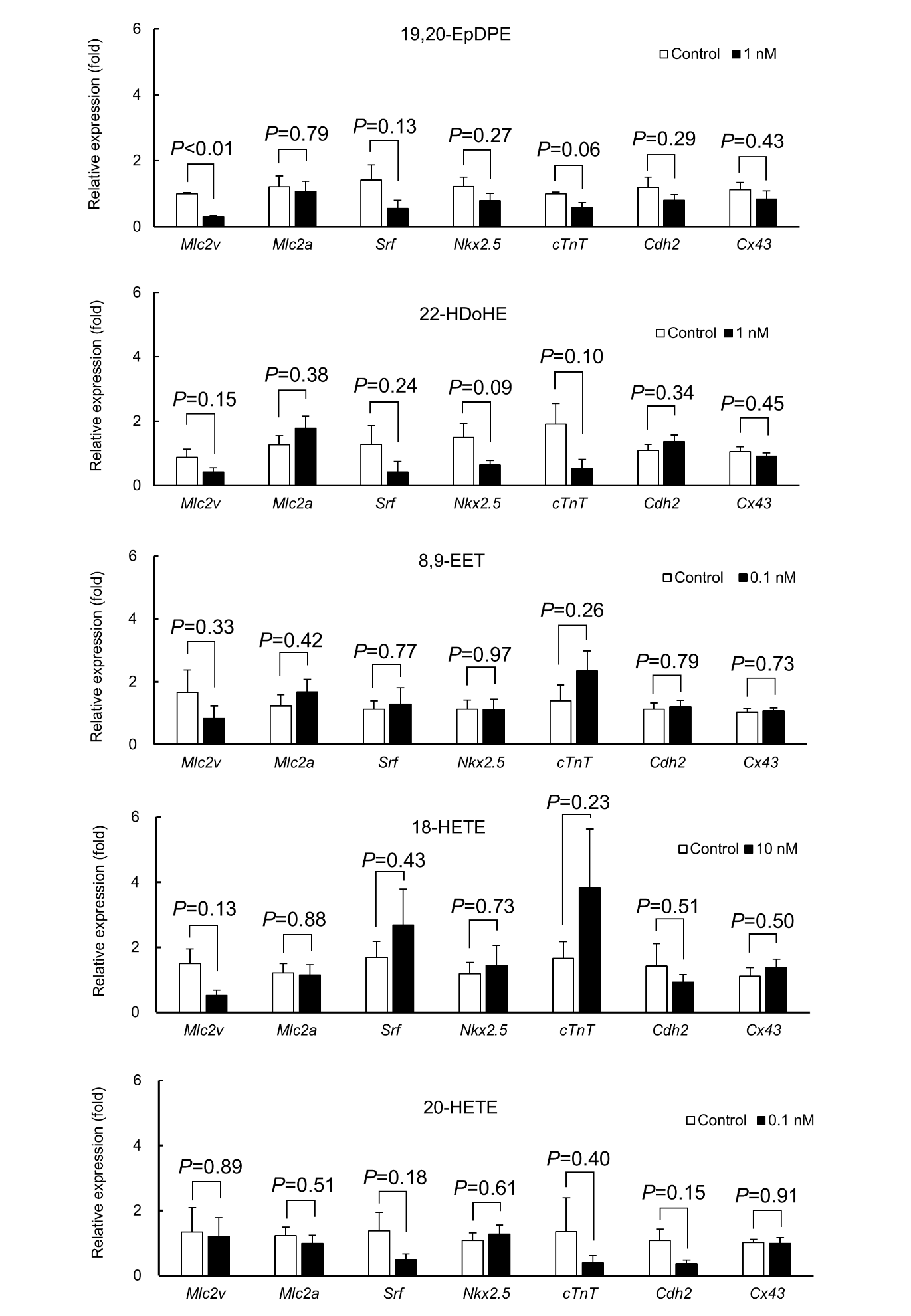


**Figure S1**: Effects of docosahexaenoic acid- and arachidonic acid-derived metabolites on mRNA expression involved in differentiation, maturation, and cell adhesion of embryonic rat cardiomyocytes. β-actin was used as an internal control. Gene expression in individual samples was determined using ΔΔCt method. Mean levels in the control group were arbitrarily assigned a value of 1. Data are expressed as mean ± SE. n=5-9.


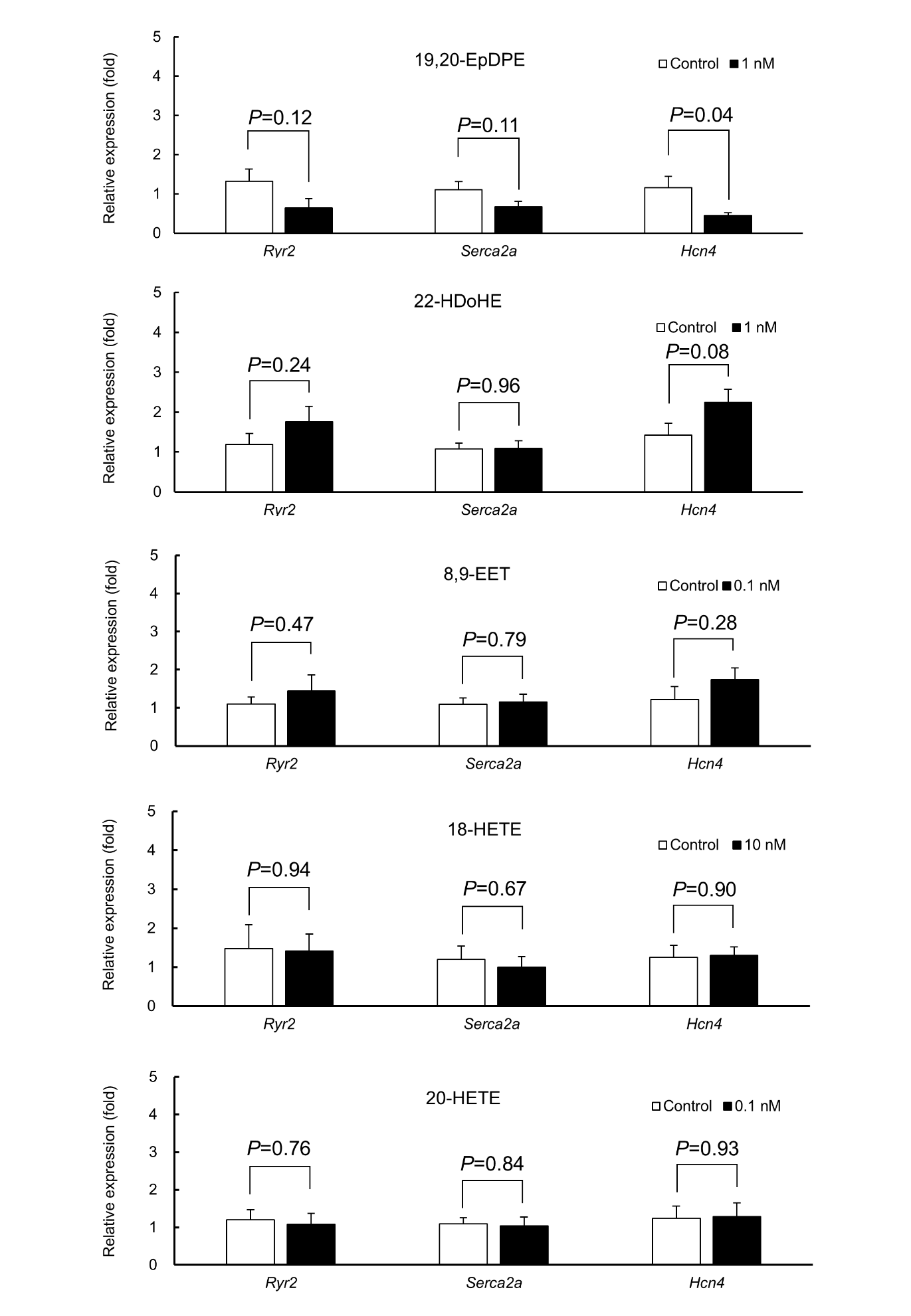


**Figure S2**: Effects of docosahexaenoic acid- and arachidonic acid-derived metabolites on mRNA expression involved in calcium transport and heart contraction of embryonic rat cardiomyocytes. β-actin was used as an internal control. Gene expression in individual samples was determined using ΔΔCt method. Mean levels in the control group were arbitrarily assigned a value of 1. Data are expressed as mean ± SE. n=5-9.


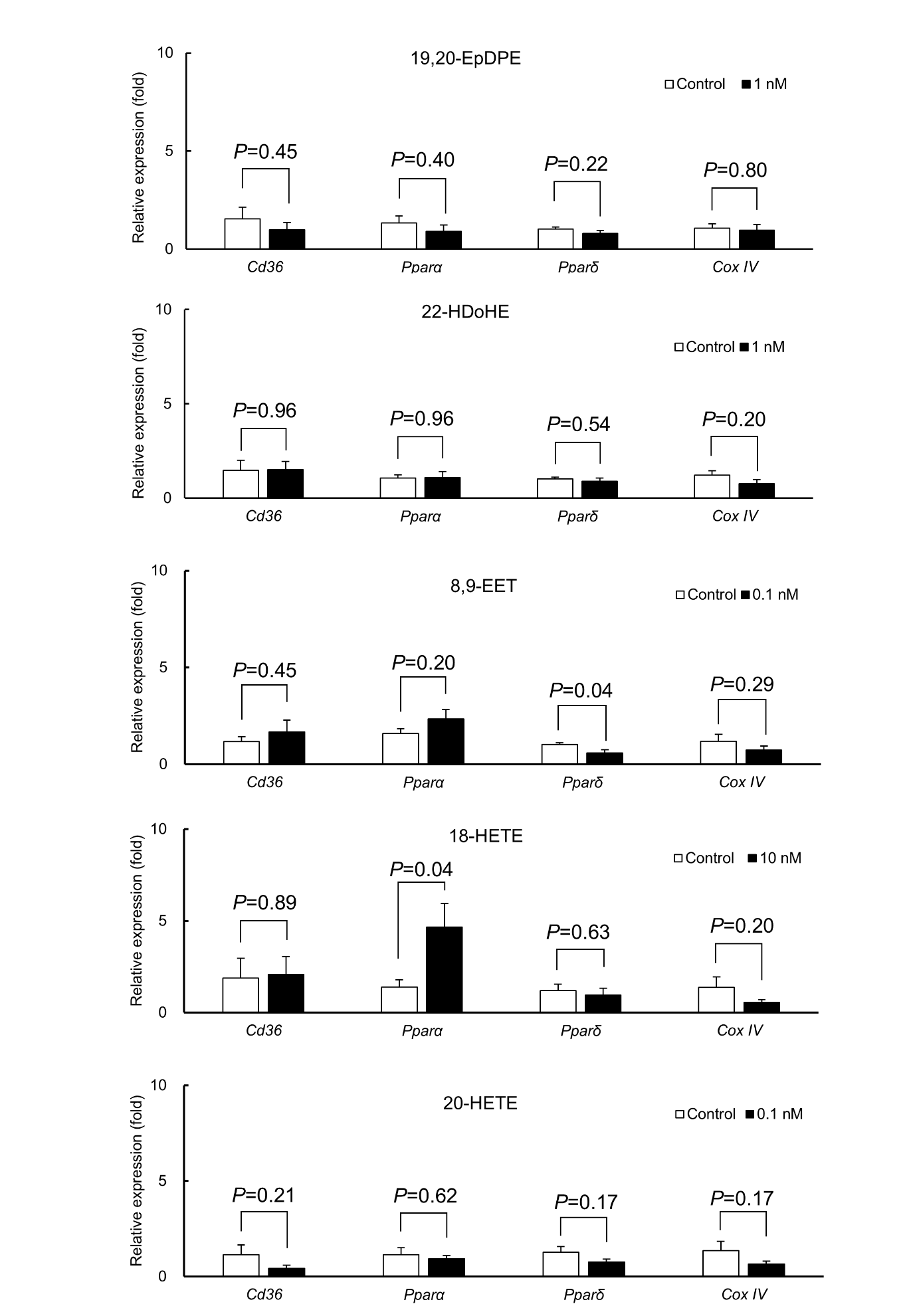


**Figure S3**: Effects of docosahexaenoic acid- and arachidonic acid-derived metabolites on mRNA expression involved in lipid metabolism of embryonic rat cardiomyocytes. β-actin was used as an internal control. Gene expression in individual samples was determined using ΔΔCt method. Mean levels in the control group were arbitrarily assigned a value of 1. Data are expressed as mean ± SE. n=5-9.
